# Evaluation of stability of directly standardized rates for sparse data using simulation methods

**DOI:** 10.1186/s12963-018-0177-1

**Published:** 2018-12-22

**Authors:** Joan K. Morris, Joachim Tan, Paul Fryers, Jonathan Bestwick

**Affiliations:** 1 Centre for Environmental and Preventive Medicine, Wolfson Institute of Preventive Medicine, Barts and the London School of Medicine and Dentistry, Queen Mary University of London, Charterhouse Square, London, EC1M 6BQ UK; 2 Health Intelligence Division, Public Health England, Wellington House, 133-155 Waterloo Road, London, SE1 8UR UK

**Keywords:** Direct standardization, Monte Carlo simulation, Confidence interval coverage, Tiwari, Dobson

## Abstract

**Background:**

Directly standardized rates (DSRs) adjust for different age distributions in different populations and enable, say, the rates of disease between the populations to be directly compared. They are routinely published but there is concern that a DSR is not valid when it is based on a “small” number of events. The aim of this study was to determine the value at which a DSR should not be published when analyzing real data in England.

**Methods:**

Standard Monte Carlo simulation techniques were used assuming the number of events in 19 age groups (i.e., 0–4, 5–9, ... 90+ years) follow independent Poisson distributions. The total number of events, age specific risks, and the population sizes in each age group were varied. For each of 10,000 simulations the DSR (using the 2013 European Standard Population weights), together with the coverage of three different methods (normal approximation, Dobson, and Tiwari modified gamma) of estimating the 95% confidence intervals (CIs), were calculated.

**Results:**

The normal approximation was, as expected, not suitable for use when fewer than 100 events occurred. The Tiwari method and the Dobson method of calculating confidence intervals produced similar estimates and either was suitable when the expected or observed numbers of events were 10 or greater. The accuracy of the CIs was not influenced by the distribution of the events across categories (i.e., the degree of clustering, the age distributions of the sampling populations, and the number of categories with no events occurring in them).

**Conclusions:**

DSRs should not be given when the total observed number of events is less than 10. The Dobson method might be considered the preferred method due to the formulae being simpler than that of the Tiwari method and the coverage being slightly more accurate.

## Background

Directly standardized rates (DSRs) are routinely produced by national organizations to compare rates, such as for diseases, across different geographic areas. They are calculated by applying the observed age specific rates in each population to a population with a standard age distribution. As the rates usually apply to specific causes of death or disease, which are generally independent (apart from, for example, contagious diseases) and occur relatively infrequently, the Poisson distribution is used to model the occurrence of such events and to derive the confidence intervals for the standardized rates. There are four different approaches to estimating the confidence intervals for these rates: (i) using the normal approximation of the Poisson distribution, (ii) treating the DSR as a weighted sum of Poisson variables, (iii) assuming a gamma distribution, or (iv) assuming a beta distribution. The three most common methods of calculating confidence intervals for directly standardized rates are, firstly, the normal approximation for the total number of events [1] used, for example, in the IARC Cancer Incidence in Five Continents [2]. Secondly, the Dobson method which is an example of treating the DSR as a weighted sum of Poisson variables [3] and used, for example, by Public Health England in Official Statistics, such as for the Public Health Outcomes Framework [4]. Thirdly the Tiwari modified gamma method [5] which uses the beta distribution and is a modification of the gamma method proposed by Fay and Feuer [6] used by the Surveillance, Epidemiology, and End Results (SEER) Program of the National Cancer Institute in Bethesda, Maryland and the Italian Association of Cancer Registries [7].

It is known that the Normal approximation is appropriate only for large numbers of events (such as those occurring in whole countries) [8]. The Stata module “distrate” for calculating confidence intervals for directly standardized rates [7, 9] provides two methods for calculating confidence intervals: the Tiwari method as the default method with the Dobson method available as an option. It states that the Tiwari method “produces valid confidence intervals even when the number of cases is very small.” The Stata manual does not specify what “small” is [9]. There is uncertainty as to how “small” is too small, with the Australian Institute of Health and Welfare and the US Centers for Disease Control and Prevention recommending below 25 events [10, 11].

However, small numbers of events often occur. For example, in the IARC Cancer Incidence in Five Continents publication, there were many cancers with less than ten cases in several populations [2]. A simulation study by Ng et al. [12] examined in detail the relative performance of many different models over several different scenarios. They concluded that the methods by Dobson and Tiwari performed the most consistently. However, their simulations were limited to only considering standardizing for six age groups, where all populations had more than 10 events and did not have widely varying age-specific event rates and age distributions. We were therefore interested in examining the sensitivity of the Dobson and Tiwari methods for fewer than 10 events occurring over 19 different age groups and with widely varying age-specific risks and population sizes. The aim of this study was to investigate the accuracy of the two methods compared with that of the normal approximation using simulation procedures which mirror a variety of plausible real-world scenarios occurring in the production of age-specific rates in England. Real data were then used to demonstrate the methods.

## Methods

### Definition of an accurate confidence interval

A confidence interval is considered “accurate” if the probability that it includes the true value (the “coverage”) is close to the stated target probability; i.e., a 95% confidence interval should include the true value approximately 95% of the time. In this analysis we defined the terms “conservative,” “liberal,” and “accurate” as follows. If the coverage exceeds the stated probability by more than 40% the confidence interval is “conservative.” If the coverage is under the stated probability by more than 40% the confidence interval is “liberal.” Otherwise, it is accurate. In other words, a 95% confidence interval is considered accurate if its coverage is between 93% (100–(1.4 × 5))% and 97 (100–(0.6 × 5))%. It is generally considered preferable for confidence intervals to be conservative rather than liberal, but that can depend on the context of the analysis and the nature of any decisions to be based on the analysis.

### Simulation methods

The number of events occurring in each of 19 5-year age specific categories (i.e., 0–4, 5–9, ... 90+ years) was estimated using the random Poisson generating function in Stata for the following scenarios and restrictions which are sufficient to uniquely specify the age specific rates (*θ*_*i*_) and the size of the exposed population in each age specific category (*d*_*i*_).

The scenarios considered and assumptions made were:

1. The size of the exposed population in each age category (*d*_*i*_) was assumed to be a linear function of age with the ratio of the population in the youngest category to that in the oldest category being 1 (all age categories the same size), 5 or 50. This is not restrictive as when calculating the DSRs the order of the age groups is unimportant. The total exposed population was 190,000, with an average of 10,000 in each category. An additional scenario assumed the sample population had the same age distribution as that of the European Standard Population.
2. The age specific rates (*θ*_*i*_) were assumed to be a linear function of age with the ratio of the rate (i.e., relative risk) in the oldest to that in the youngest being 1 (no association with age), 50, 500, or 5000. This is not restrictive as when calculating the DSRs the order of the age groups is unimportant.
3. The total expected number of events (∑*d*_*i*_*θ*_*i*_) that would occur across all age groups was specified as: 1,2,3,4,5,6,7,8,9,10,11,12,13,14,15,25 and 100.

For each scenario the observed DSRs were calculated using formula A below. The 95% confidence intervals were calculated for the normal approximation method, the Dobson method [3] and Tiwari method [5] using the formulae B, C, and D below. This was repeated to generate 10,000 sets of data. For each scenario:

1. the true DSR ($\frac{1}{\sum {w}_i}\sum {w}_i{\theta}_i$) was calculated using the age specific rates (*θ*_*i*_) and the European Standard Population weights (*w*_*i*_)
2. the observed DSRs and confidence intervals were divided by the true DSR to enable simple comparisons between the scenarios to be made
3. the inclusion of 1 within the specified confidence intervals was noted
4. the variation of (European standard population/sample population) divided by sum of (European standard population/sample population)^2^ was calculated as a measure of how much the sample population differs from the European standard population.

Stata Version 14 was used to perform all simulations [9].

#### Calculating a directly standardized rate (DSR)

The DSR, *R*, is calculated as a weighted average of *n* age-specific rates $\left(\frac{x_i}{d_i}\right)$:

$$ R=\frac{1}{\sum {w}_i}\sum \frac{w_i{x}_i}{d_i} $$

Where

*x*_*i*_ are the observed age-specific numerator events;

*d*_*i*_ are the age-specific denominator populations;

*w*_*i*_ are taken from the 2013 European Standard Population (ESP):

**Table:** 

Age group	0–4	5–9	10–14	15–19	20–24	25–29	30–34	35–39	40–44	45–49
2013 ESP	5000	5500	5500	5500	6000	6000	6500	7000	7000	7000

**Table:** 

Age group	50–54	55–59	60–64	65–69	70–74	75–79	80–84	85–89	90+
2013 ESP	7000	6500	6000	5500	5000	4000	2500	1500	1000

#### The normal approximation for calculating confidence intervals [4]

The 100(1-α)% lower and upper confidence limits, LCL and UCL, are defined as:

$$ LCL=R-\sqrt{\frac{\sum \frac{w_i^2\ {x}_i}{{d_i}^2}}{\sum {x}_i{\left(\sum {w}_i\right)}^2}}\ \left({Z}_{1-\raisebox{1ex}{$\alpha $}\!\left/ \!\raisebox{-1ex}{$2$}\right.}\right) $$

$$ UCL=R+\sqrt{\frac{\sum \frac{w_i^2\ {x}_i}{{d_i}^2}}{\sum {x}_i{\left(\sum {w}_i\right)}^2}}\ \left({Z}_{1-\raisebox{1ex}{$\alpha $}\!\left/ \!\raisebox{-1ex}{$2$}\right.}\right) $$

Where

*R*, *x*_*i*_, *d*_*i*_ and *w*_*i*_ are defined as in A.

$$ {Z}_{1-\raisebox{1ex}{$\alpha $}\!\left/ \!\raisebox{-1ex}{$2$}\right.} $$ is the $$ 100\left(1-\frac{\alpha }{2}\right) $$th percentile value of the inverse standard normal distribution.

#### The Dobson method of calculating confidence intervals [3]

The 100(1 − *α*)% lower and upper confidence limits, *LCL* and *UCL*, are defined as:

$$ LCL=R+\sqrt{\frac{\sum \frac{w_i^2\ {x}_i}{{d_i}^2}}{\sum {x}_i{\left(\sum {w}_i\right)}^2}}\left(\frac{Inv{\mathrm{X}}^2\left(1-\frac{\alpha }{2},2\sum {x}_i\right)}{2}-\sum {x}_i\right) $$

$$ UCL=R+\sqrt{\frac{\sum \frac{w_i^2\ {x}_i}{{d_i}^2}}{\sum {x}_i{\left(\sum {w}_i\right)}^2}}\left(\frac{Inv{\mathrm{X}}^2\left(\frac{\alpha }{2},2\sum {x}_i+2\right)}{2}-\sum {x}_i\right) $$

Where

*R*, *x*_*i*_, *d*_*i*_ and *w*_*i*_ are defined as in A.

*Inv* Χ^2^(*π*, *ν*) is the 100(1 − *π*)th percentile value of the inverse chi-squared distribution with *ν* degrees of freedom

#### The Tiwari modified gamma method of calculating confidence intervals [5]

The 100(1 − *α*)% lower and upper confidence limits, *LCL* and *UCL*, are defined as:

$$ LCL=\frac{\sum \frac{w_i^2\ {x}_i}{{d_i}^2}}{2R{\left(\sum {w}_i\right)}^2}\  Inv\ {\mathrm{X}}^2\left(1-\frac{\alpha }{2},\frac{2{R}^2{\left(\sum {w}_i\right)}^2}{\sum \frac{w_i^2\ {x}_i}{{d_i}^2}}\right) $$

$$ UCL=\frac{\sum \frac{w_i^2\ {x}_i}{{d_i}^2}+\frac{1}{n}\sum \frac{{w_i}^2}{{d_i}^2}}{2{\left(\sum {w}_i\right)}^2\left(R+\frac{1}{n\sum {w}_i}\sum \frac{w_i}{d_i}\right)}\  Inv\ {\mathrm{X}}^2\left(\frac{\alpha }{2},\frac{2{\left(R+\frac{\sum \frac{w_i}{d_i}}{n\sum {w}_i}\right)}^2{\left(\sum {w}_i\right)}^2}{\sum \frac{w_i^2\ {x}_i}{{d_i}^2}+\frac{1}{n}\sum \frac{{w_i}^2}{{d_i}^2}}\right) $$

Where

*R*, *x*_*i*_, *d*_*i*_ and *w*_*i*_ and *Inv* Χ^2^(*π*, *ν*) are defined as in A and C

### Analysis of real data

The numbers of suicides occurring from 2013 to 2015 in the 326 local authority districts in England for males and females were analyzed using the procedure “distrate” in Stata Version 14. In each district the gender-specific DSRs were calculated using the 2013 European Standard Population weights and the Tiwari method to calculate the 95% confidence intervals. In addition, using the European Standard Population, the overall standardized rates for England were calculated for males and females separately. Suicides were chosen for the analysis because of the small numbers of deaths occurring at district level.

## Results

Table 1 gives the results of the simulations for 30 scenarios: (Expected total number of events: 5,10,15,25 and 100) x (Sample populations: all age groups same size, 50 times larger in youngest compared with oldest, same distribution as European standard population) x (Relative risk in oldest vs. youngest: one and 5000).

**Table 1:** Empirical results from 10,000 simulations of weighted sums of Poisson parameters. (EU = distribution of sample population is same as European standard population)

			Normal approximation			Dobson method			Tiwari method		
Expected events	Population ratio youngest vs. oldest	Incidence ratio oldest vs. youngest	95% LCL	95% UCL	Coverage (%)	95% LCL	95% UCL	Coverage (%)	95% LCL	95% UCL	Coverage (%)
5	1	1	0.05	1.9	89.9	0.27	2.3	96.4	0.28	2.3	97.0
5	1	5000	0.02	1.9	87.6	0.25	2.4	94.6	0.26	2.4	96.9
5	50	1	0.03	1.9	87.0	0.24	2.4	94.0	0.26	2.6	97.9
5	50	5000	0.06	1.8	89.2	0.27	2.3	95.5	0.28	2.4	97.9
5	EU	1	0.12	1.9	86.9	0.32	2.3	98.0	0.32	2.3	98.0
5	EU	5000	0.12	1.9	87.0	0.32	2.3	97.9	0.32	2.3	97.9
10	1	1	0.34	1.6	92.2	0.44	1.9	96.3	0.45	1.9	96.4
10	1	5000	0.30	1.7	92.2	0.41	1.9	96.1	0.43	1.9	96.6
10	50	1	0.30	1.6	91.2	0.41	1.9	95.4	0.42	2.0	97.7
10	50	5000	0.33	1.6	92.0	0.43	1.9	95.8	0.44	1.9	96.6
10	EU	1	0.38	1.6	92.7	0.48	1.8	97.5	0.48	1.8	97.5
10	EU	5000	0.38	1.6	92.6	0.48	1.8	97.6	0.48	1.8	97.6
15	1	1	0.46	1.5	93.2	0.53	1.7	96.1	0.53	1.7	96.2
15	1	5000	0.43	1.5	93.3	0.51	1.7	96.0	0.51	1.7	96.3
15	50	1	0.43	1.5	92.1	0.50	1.7	95.6	0.51	1.8	97.0
15	50	5000	0.45	1.5	92.5	0.52	1.7	96.2	0.52	1.7	96.6
15	EU	1	0.49	1.5	91.8	0.56	1.6	96.3	0.56	1.6	96.3
15	EU	5000	0.49	1.5	92.0	0.56	1.6	96.3	0.56	1.6	96.3
25	1	1	0.58	1.4	93.9	0.62	1.5	95.9	0.62	1.5	96.0
25	1	5000	0.56	1.4	93.9	0.61	1.5	96.0	0.61	1.5	96.1
25	50	1	0.56	1.4	93.4	0.60	1.5	95.5	0.61	1.5	96.4
25	50	5000	0.57	1.4	93.5	0.62	1.5	95.6	0.62	1.5	95.9
25	EU	1	0.61	1.4	94.7	0.65	1.5	95.5	0.65	1.5	95.5
25	EU	5000	0.61	1.4	94.8	0.65	1.5	95.5	0.65	1.5	95.5
100	1	1	0.79	1.2	94.9	0.80	1.2	95.6	0.80	1.2	95.6
100	1	5000	0.78	1.2	95.0	0.79	1.2	95.8	0.79	1.2	95.8
100	50	1	0.78	1.2	94.5	0.79	1.2	95.4	0.79	1.2	95.7
100	50	5000	0.79	1.2	94.9	0.80	1.2	95.8	0.80	1.2	95.8
100	EU	1	0.80	1.2	94.6	0.81	1.2	95.5	0.81	1.2	95.5
100	EU	5000	0.80	1.2	94.5	0.81	1.2	95.4	0.81	1.2	95.4

**Fig. 1:**
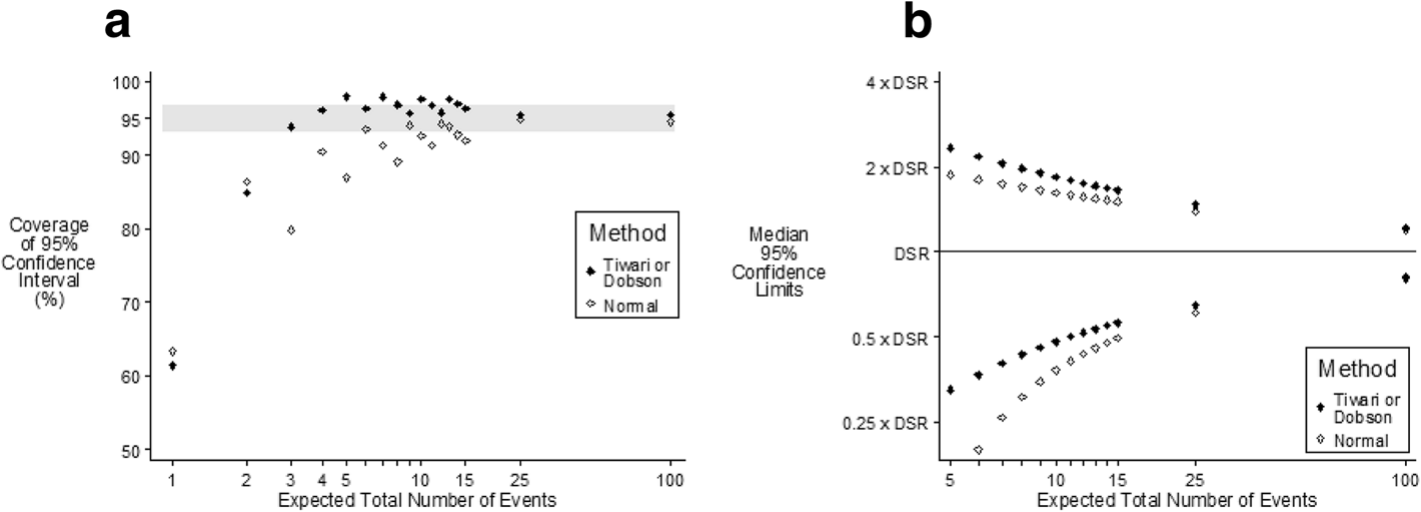
95% confidence intervals of DSR according to method of calculation when the relative risk in the oldest vs. youngest age is 5000 and the sample population has the same age distribution as the European standard population. [Grey band indicates “Accurate Coverage”]. **a** Coverage and **b** Median values

**Fig. 2:**
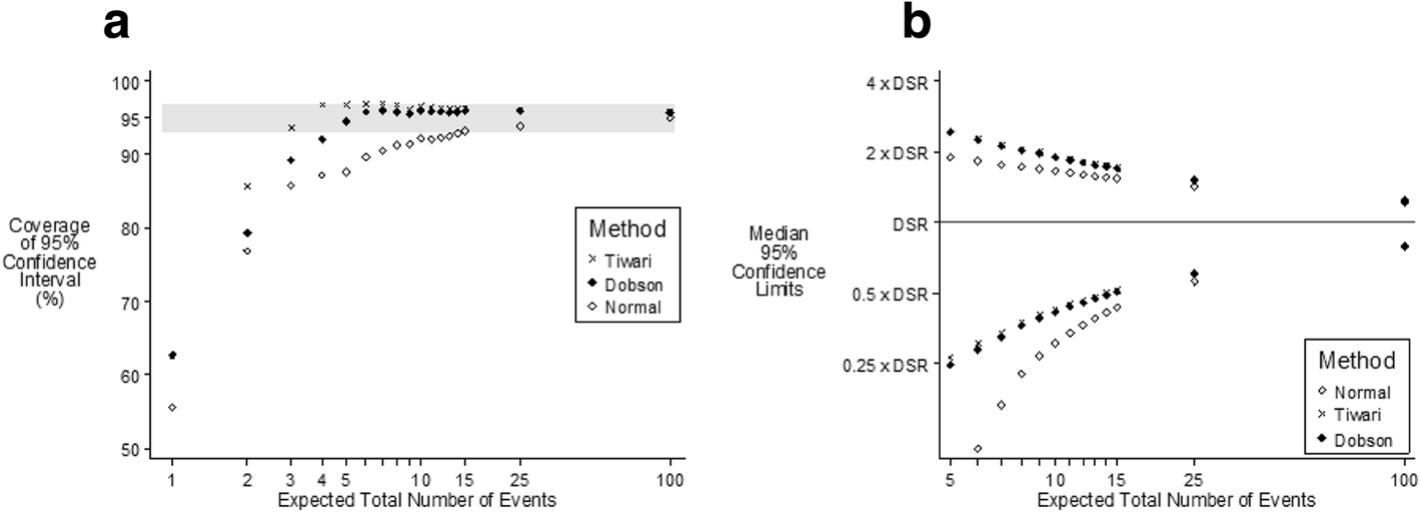
95% confidence intervals of DSR according to method of calculation when the relative risk in the oldest vs youngest age is 5000 and the number of people is the same for each age in the sample population. [Grey band indicates “Accurate Coverage”]. **a** Coverage and **b** Median values

The Dobson and Tiwari methods differ in the method of adjusting for the effect of differing weights given to each age specific rate, with the Tiwari method in effect having a greater adjustment for the different weights. Both methods give identical confidence intervals and hence identical coverage when there is no weighting (the scenario in which the age distribution in the sample is the same as that in the standardized population) as shown in Fig. 1. For all three methods with very small numbers of events the coverage does not always improve with increasing sample size. This is because of the discrete nature of the data with only integer counts being able to occur. The coverage illustrated in Fig. 1 for a relative risk in the oldest age group vs. the youngest age group of 5000 is not materially different from that when the incidence is the same for all age groups (Table 1). Figure 2a shows that when the sample population has an equal number of people in each age group (very different from the standard population) and when the relative risk in the oldest age group vs. the youngest age group is 5000 the Tiwari method is able to provide more accurate coverage when the expected number of events is less than five, with both Dobson and Tiwari having accurate coverage when the expected number of events is six or more.

Figure 2b plots the median values of the upper and lower 95% confidence limits and shows that the Dobson and Tiwari methods are very similar, with the Normal approximation predicting both the upper and lower confidence limits to be lower than those predicted by the other methods. In addition, using the Normal approximation to calculate the lower 95% confidence interval occasionally resulted in incorrect negative values being predicted.

**Fig. 3:**
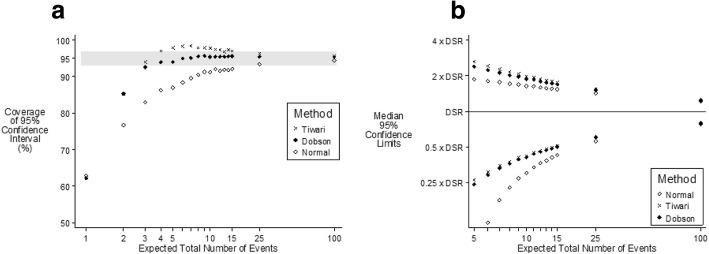
95% confidence intervals of DSR according to method of calculation when the risk Is independent of age and the sample population is 50 times larger in the youngest compared to oldest age group. [Grey band indicates “Accurate Coverage”]. **a** Coverage and **b** Median values

In the situation where the youngest age groups have sample populations 50 times greater than the oldest age groups and the relative risks are the same in all age groups (Fig. 3), the Tiwari method will overcompensate for the differing weights (compensation is unnecessary as there is no age effect). Dobson is accurate for four or more expected number of events and Tiwari will be slightly conservative for fewer than 15 observations and accurate for 15 or more observations.

**Fig. 4:**
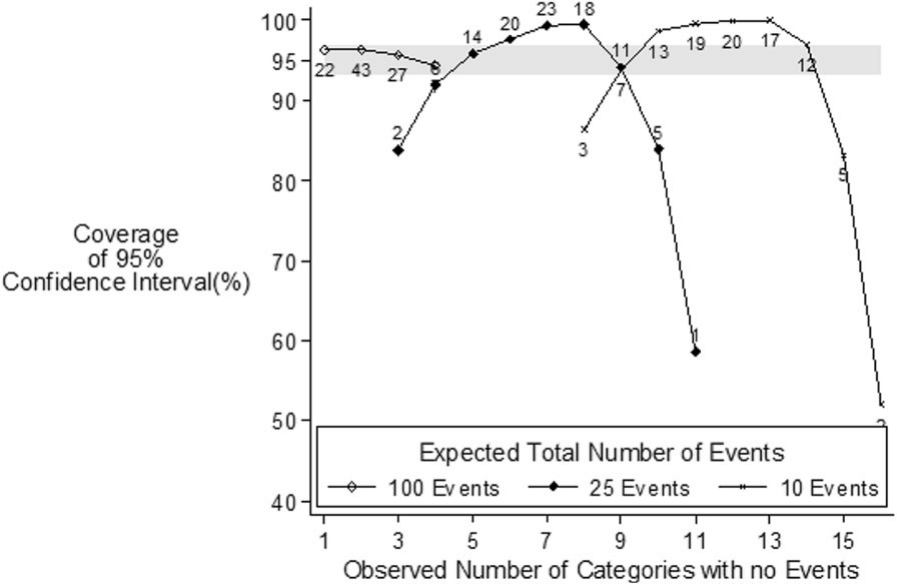
Coverage of 95% confidence intervals according to the observed number of categories with no events [Numerical labels denote relative occurrence] and the total expected number of events for the Dobson method when the incidence in the oldest age is 500 times the incidence in the youngest age and the number of people is the same for each age in the sample population. [Grey band indicates “Accurate Coverage”] Rare scenarios, i.e., occurring < 1% of the time, have not been plotted

The effect of varying the distribution of incidence by age was examined by simulating data with the incidence ratio (oldest:youngest) varying from one (the events being evenly distributed across all categories) to 5000 (the events being strongly clustered towards the older ages). The effect of the degree of clustering was also examined by looking at the number of categories in which no events occurred (Fig. 4) using the Dobson method with the ratio of the incidence in the oldest compared with youngest category being 500. When 100 events are expected amongst 19 categories the lowest two groups are not expected to have any events occurring in them. This happened in 43% of all simulations. Seven percent of the simulations had four categories with zeros – even when this happened the estimated confidence intervals were “accurate.” As the total number of events decreases the expected numbers of categories with zero events increases and therefore having a large number of zero categories does not automatically result in 95% CIs that do not reach the nominal coverage level (i.e., that are too liberal). In Fig. 4 the coverage appears to be lower when there are either a relatively high number of categories with zero events or a relatively low number of such categories. In fact, these situations occur rarely (the numbers on the graph indicate the relative frequency) and therefore it is the rarity of the situation that is associated with a low coverage rather than the number of categories per se with no events in them. The smaller the total number of events expected the greater the occurrence of extreme simulations (for 10 events, 10% of simulations had “inaccurate” 95% CIs whereas < 1% of simulations for 100 events had “inaccurate” 95% CIs).

In Figs. 1, 2, and 3 both Dobson and Tiwari provide either accurate or conservative 95% confidence intervals when the expected number of events is five or more. However, in most situations the expected number of events is unknown and all that is known is the observed number of events. Using the simulated data, Table 2 shows that 10 or more events are observed < 0.1% of the time if the expected number of events is < 3, 0.1% if the expected number is three and only 0.8% if the expected number is four. This means that if 10 or more events are observed the expected number of events is unlikely to be below five. Similarly if eight or more events are observed then 6.4% of the time the expected number is less than five, which is judged to be a relatively common occurrence. If nine events are observed then 2.4% of the time the expected number of events is less than five. The authors judge that it would be more cautious to insist that at least 10 events are observed, but clearly there could be situations were nine events might be considered a more appropriate cut-off. Therefore, if the observed number of events is 10 or more the 95% confidence interval is very likely to be “accurate.” Figures 2b and 3b show that when the expected total number of events was 10 or more, the upper confidence limit was slightly more than twice the DSR and the lower confidence limit was slightly less than 50% of the DSR. The median width of the confidence interval for 10 expected events is identical to the median width for 10 observed events. Therefore if 10 events are observed the 95% confidence interval is very likely to be accurate.

**Table 2:** The proportion of times the observed number of events or more occurred according to the expected number of events when the incidence in the oldest age group is 500 times the incidence in the youngest age group

Observed number of events	Expected number of events				
	1	2	3	4	5
	Proportion of times the observed number of events or more occurred				
None	100.0	100.0	100.0	100.0	100.0
1	63.1	86.9	95.1	98.3	99.5
2	26.8	60.0	79.8	91.2	96.2
3	7.8	33.1	57.3	76.5	88.1
4	1.8	14.7	35.1	56.7	73.2
5	0.3	5.5	18.1	36.8	55.5
6	0.0	1.6	7.9	21.3	38.3
7	0.0	0.5	3.2	10.7	24.5
8		0.1	1.4	4.9	13.7
9		0.0	0.3	2.1	7.2
10		0.0	0.1	0.8	3.3
11		0.0	0.0	0.3	1.5
12			0.0	0.1	0.6
13			0.0	0.0	0.2
14				0.0	0.1
15				0.0	0.0
16					0.0

### Real data analysis

Table 3 shows that many of the local authority districts in England had very small numbers of suicides, particularly amongst females. Out of the 326 districts there were at least 10 male suicides occurring in 317 (97%) and at least 10 female suicides occurring in only 141 (43%). In those cases the confidence intervals calculated by the Dobson and Tiwari methods agreed to within +/− 1% for both the lower and upper bounds. For the cases with fewer than 10 suicides, as expected, the confidence intervals were less consistent with only around half agreeing to within 1%.

**Table 3:** Number of suicides in each district by gender

Numbers of suicides	Number of districts	
	Female	Male
0	1	0
1	5	1
2	15	0
3	19	1
4	21	1
5	27	0
6–9	97	6
10–19	110	80
20–49	28	178
50–99	3	51
100+	0	8
Total	326	326

Amongst the nine districts in which fewer than 10 suicides in men were observed there were only two districts where the confidence interval (consistent for both methods) did not include the DSR for England of 1.58 per 10,000 (six and nine suicides in these two districts). Similarly amongst the 185 districts in which fewer than 10 suicides in women were observed there were only three districts where the confidence interval (consistent for both methods) did not include the DSR for England of 0.47 per 10,000 (one, one, and three suicides in these districts). In routine publication of DSRs the confidence intervals are presented to aid interpretation, with the guidance being that districts where the 95% CI includes the England comparator value should not assume there is any underlying reason for the difference as it is likely to be due to chance variation. For these data there are therefore only two districts in which this assumption may be misleading for the male suicide rates. For females the numbers of counts are so small that the confidence intervals are not reliable in many districts.

## Discussion

The strength of this study is that it is based on realistic simulated data. Firstly, as is standard practice, the European Standard Population weights for 19 5-year age groups were used. Secondly, the age specific rates were allowed to vary by as much as 5000 times for the oldest compared with the youngest age group. These variations do occur in real observed data: for example, the age specific rate of stroke deaths in men aged 90+ is over 5000 times that in men aged 15–19 [13]. Thirdly, the age distributions in the samples were allowed to vary considerably from the European Standard Population. This variation is measured by calculating the standardized variation of the ratio of the European Standard Population over the sample population for each age group (see methods section). The standardized variation ranged from 0 (for the scenario with the sample population having the same distribution as the standard population) to 0.00108 (ratio of size of the largest to smallest sample age group is equal to five) and 0.0133 (ratio of size of the largest to smallest sample age group is equal to 50). The observed variations in 326 local authority districts in England for males and females from 2013 to 2015 were from 0.0000183 to 0.00175, smaller than the most extreme scenarios modeled.

In all the simulations the total population size was 190,000. This is similar to 170,000, which is the average population size in the 326 local authority districts in England and also allows 10,000 people in each of the 19 age groups. The results are not sensitive to this assumption.

A limitation of this analysis is that it depends on the assumption that the occurrence of an event follows a Poisson distribution. Poisson distributions assume events are independent, an infinite number of events may occur over a long period of time, and that events occur only rarely in short periods of time. For most diseases and causes of death event independence is likely to be a reasonable assumption. It will not apply to rates of contagious diseases, for example, where the occurrences are not independent, or where extreme weather or events cause spikes in deaths from a single external factor. However, these confidence interval methods should not be used in such cases. The assumption of an infinite number of events occurring over a long period of time is also generally reasonable as for example when analysing a specific cancer the size of the population (i.e., the total number of deaths that could occur) is considerably greater than the numbers of actual deaths likely to occur and can therefore be thought of as infinite.

As stated in the methods section, specifying linear functions for the incidence and the population sizes is not restrictive, because when calculating the DSRs the order of the age groups is irrelevant. The effect of altering the incidence and age association is to create different amounts of clustering within the numbers of observed events; a ratio of 5000:1 in incidence between the highest and lowest categories will result in the lowest categories having very few events in contrast to a ratio of 1 which will spread the events evenly.

Both the Dobson and the Tiwari methods of calculating confidence intervals are influenced by the variation of the ratio of the population years in the standard population to the population years in the sample. Ng et al. [12] specified that if this variation was small (< 0.01 – as is the case in most of our modeled scenarios and the real data) then the Tiwari method was the recommended method if it was acceptable that the coverage was above the nominal value – as has been seen in these simulations. Ng et al. recommended the original gamma method proposed by Fay and Feuer [6] if one is more concerned with a symmetrical confidence interval that is closer to the nominal coverage.

The normal approximation for calculating confidence intervals was included in this study in order to provide a benchmark against which to judge the Dobson and Tiwari methods. In practice if the normal approximation was the method of choice then the use of continuity corrections to improve the fit to the normal distribution would need to be investigated [14], such as that suggested by Begaud et al. [15]. However as even standard spreadsheet packages can calculate the chi-squared distribution and can therefore calculate confidence intervals using both Dobson and Tiwari methods, efforts to improve the normal approximation were not considered further.

In agreement with Ng et al. [12] the coverage of the Dobson and Tiwari methods are both considered accurate for 10 or more observed events. Coverage from the Dobson method is consistently closer to 95%, with the coverage from the Tiwari method tending to be above 95%. However, as the confident limits from the two methods differ by less than 0.1% the differences in estimates are not significant.

## Conclusion

The results from this simulation confirm those predicted from other studies [3, 12, 15] and lead to the recommendation that at least 10 events must have occurred for a directly standardized rate to be published. Both the Dobson and Tiwari methods produce “accurate” confidence intervals when 10 or more events are observed. As expected, the normal approximation should not be used for fewer than 25 events. The Dobson method might be considered the preferred method due to the formulae being simpler than that of the Tiwari method and the coverage being slightly closer to 95%.
